# High expression of TMEM180, a novel tumour marker, is associated with poor survival in stage III colorectal cancer

**DOI:** 10.1186/s12885-021-08046-6

**Published:** 2021-03-23

**Authors:** Takuya Shiraishi, Koji Ikeda, Yuichiro Tsukada, Yuji Nishizawa, Takeshi Sasaki, Masaaki Ito, Motohiro Kojima, Genichiro Ishii, Ryo Tsumura, Sinji Saijou, Yoshikatsu Koga, Masahiro Yasunaga, Yasuhiro Matsumura

**Affiliations:** 1 Department of Colorectal Surgery, National Cancer Center Hospital East, 6-5-1 Kashiwanoha, Kashiwa, Chiba, 277-8577 Japan; 2 Department of General Surgical Science, Gunma University Graduate School of Medicine, 3-39-22 Showa-machi, Maebashi, Gunma 371-8511 Japan; 3 Pathology Division, National Cancer Center Hospital East, Kashiwa, 6-5-1 Kashiwanoha, Kashiwa, Chiba, 277-8577 Japan; 4 Division of Developmental Therapeutics, Exploratory Oncology Research & Clinical Trial Center, National Cancer Center, 6-5-1 Kashiwanoha, Kashiwa, Chiba, 277-8577 Japan; 5 Laboratory of RIN Institute Inc. 4th floor of National Cancer Center, Research Institute, 5-1-1 Tsukiji, Tokyo, 104-0045 Japan; 6 Department of Immune Medicine, National Cancer Center Research Institute, 5-1-1 Tsukiji, Chuo-ku, Tokyo, 104-0045 Japan

**Keywords:** Colorectal cancer, Prognosis, Transmembrane protein, TMEM180, Tumour marker

## Abstract

**Background:**

Transmembrane protein 180 (TMEM180) is a newly identified colorectal cancer (CRC)-specific molecule that is expressed very rarely in normal tissue and up-regulated under hypoxic conditions. We developed a monoclonal antibody (mAb) against TMEM180 and decided to examine the medical significance using the mAb.

**Methods:**

A total of 157 patients (86 men and 71 women; median age 63.0 years) with stage III CRC who underwent curative surgery were analyzed for TMEM180 expression as a retrospective cohort design. Immunohistochemistry with anti-TMEM180 mAb was conducted on frozen sections, and the data were evaluated for any correlation with clinicopathological indices or prognosis. SW480 CRC cells were examined to investigate the relationship between the expression of TMEM180 and tumourigenesis of xenografts.

**Results:**

In total, 92 cases had low TMEM expression and 65 had high TMEM180 expression. For disease-free survival, hazard ratio in high-TMEM180 cases was 1.449 (95% confidential interval = 0.802–2.619) higher than in low-TMEM180 cases, but the difference was not significant (*p* = 0.219). For cancer specific survival, hazard ratio in high-TMEM180 cases was 3.302 (95% confidential interval = 1.088–10.020), significantly higher than in low-TMEM180 cases (*p* = 0.035). In an assay examining in vitro colony-forming activity in soft agar, SW480-WT cells clearly formed colonies, but neither KD1 nor KD2 cells did. The in vivo tumour-initiating activity of SW480 cell lines was positively correlated with the level of TMEM180 expression.

**Conclusion:**

These results indicate that TMEM180 is a useful marker for clinical prognosis in patients with CRC. We believe that these fundamental data warrant further basic and translational studies of TMEM180, and its mAb, for development of therapeutics against CRC.

## Background

Colorectal cancer (CRC) remains one of the leading causes of cancer deaths in men and women around the world [1]. The standard treatment for patients with stage I–III CRC is surgical resection. Stage III CRC is defined as a tumour with lymph node metastasis, but no distant metastasis. Adjuvant chemotherapy has proven useful in stage III CRC patients [2], but the recurrence rates of stage III CRC patients who undergo such treatment following surgery remains about 30% [3]. Therefore, to improve the prognosis of CRC, many prognostic biomarkers associated with outcomes in patients with CRC had been explored. The poor prognosis predictive biomarkers are likely to be of great value especially if associated with novel and effective treatments.

In this context, the identification of prognostic markers that is related with effective treatment is an urgent task for reduction of CRC mortality. Previously, we found that normal colonocytes exfoliated in stool [4]. Based on this fact, we subjected 100% pure normal colonocytes obtained from lavage solution after colonoscopy to comprehensive expression analysis, and compared them to similar data from colorectal cancer cell lines. Subsequently, we conducted RT-PCR and in situ hybridization. The results of these analyses identified transmembrane protein 180 (TMEM180), a CRC-specific multi-pass membrane protein of unknown function. According to databases and our experiments, it is clear that normal organs express this molecule very rarely if at all [5]. Thus, TMEM180 may have an important function in cancer. Our previous study suggested that TMEM180 plays an important role in the uptake or metabolism of glutamine and arginine to support tumour growth and proliferation [5]. Moreover, TMEM180 appeared to be up-regulated under hypoxic conditions. Very recently, we examined the topology of this membrane protein. We determined that it has 12 transmembrane domains, and that its N- and C-termini are exposed extracellularly. At the same time, we found that the TMEM180 molecule is also present in the Golgi (data not published). We also found that the putative cation-binding site of TMEM180 is conserved among orthologs, and that its position is similar to that of the melibiose transporter MelB [6]. These findings suggest that TMEM180 acts as a cation symporter. Given these results, TMEM 180 is useful for diagnosing and treating CRC, and we believe that it is important to clarify the clinicopathological relationship between TMEM180 expression and prognosis in CRC patients.

The aim of this study was to investigate the relationship between TMEM180 expression and prognosis in stage III CRC patients. In addition, we conducted in vitro and in vivo studies using a CRC cell line in order to clarify the relationship between TMEM180 expression and tumour growth. The results revealed that TMEM180 has clinical and prognostic significance, and therefore represents a novel therapeutic target for management of CRC.

## Methods

### Patients and tumour tissues

We consecutively collected CRC patients who underwent radical surgery from January 2009 to July 2013 at the National Cancer Center Hospital East. Because our anti-TMEM180 monoclonal antibody (mAb) can be used only in frozen sections, we included the patients who were obtained surgically resected frozen tissue specimens. The tissues were resected 5–10 mm in size and collected by pathologists after surgery before fixing the specimen, and the tissues were immediately frozen. We included pathological stage III according to UICC staging criteria, and we excluded the patients who could not obtain consent. Data of clinicopathological characteristics such as age, gender, invasion depth, the number of lymph node metastases, and pathological stage were extracted from medical and surgical reports. The 7th edition UICC TNM classification was used. Postoperative follow-up was performed for patients to confirm local or distant metastasis by laboratory tests and radiological examination. The patients were followed until the final outpatient visit or until June 2018, when the data were collected. Cancer specific survival (CSS) was defined as the interval between surgery and mortality from CRC or the last follow-up. Disease-free survival (DFS) was defined as the interval between surgery and the date of recurrence or the last follow-up. This study was approved by our institutional review board (National Cancer Center Hospital; no. K2012–001). All methods were carried out in accordance with relevant guidelines and regulations, also informed consent from patients.

### Immunohistochemistry (IHC) for clinical sample

Sections of 6-μm thickness were cut from all samples. Sections were fixed with 4% paraformaldehyde at room temperature for 20 min, and then incubated with 0.3% hydrogen peroxidase at room temperature for 30 min. To prevent nonspecific staining, the sections were pre-incubated with 5% skim milk at room temperature for 60 min. Anti-TMEM180 antibody was labeled with HRP using the Peroxidase Labeling Kit-SH (Dojindo, Kumamoto City, Japan). The sections were incubated with HRP-conjugated anti-TMEM180 antibody at room temperature for 60 min. 3,3′-diaminobenzidine tetra-hydrochloride (liquid DAB+; Dako North America, Carpinteria, CA, USA) was used to reveal antigen–antibody reactions. Following counterstaining with hematoxylin, the sections were dehydrated and mounted.

### Evaluation of TMEM180 expression

**Fig. 1:**
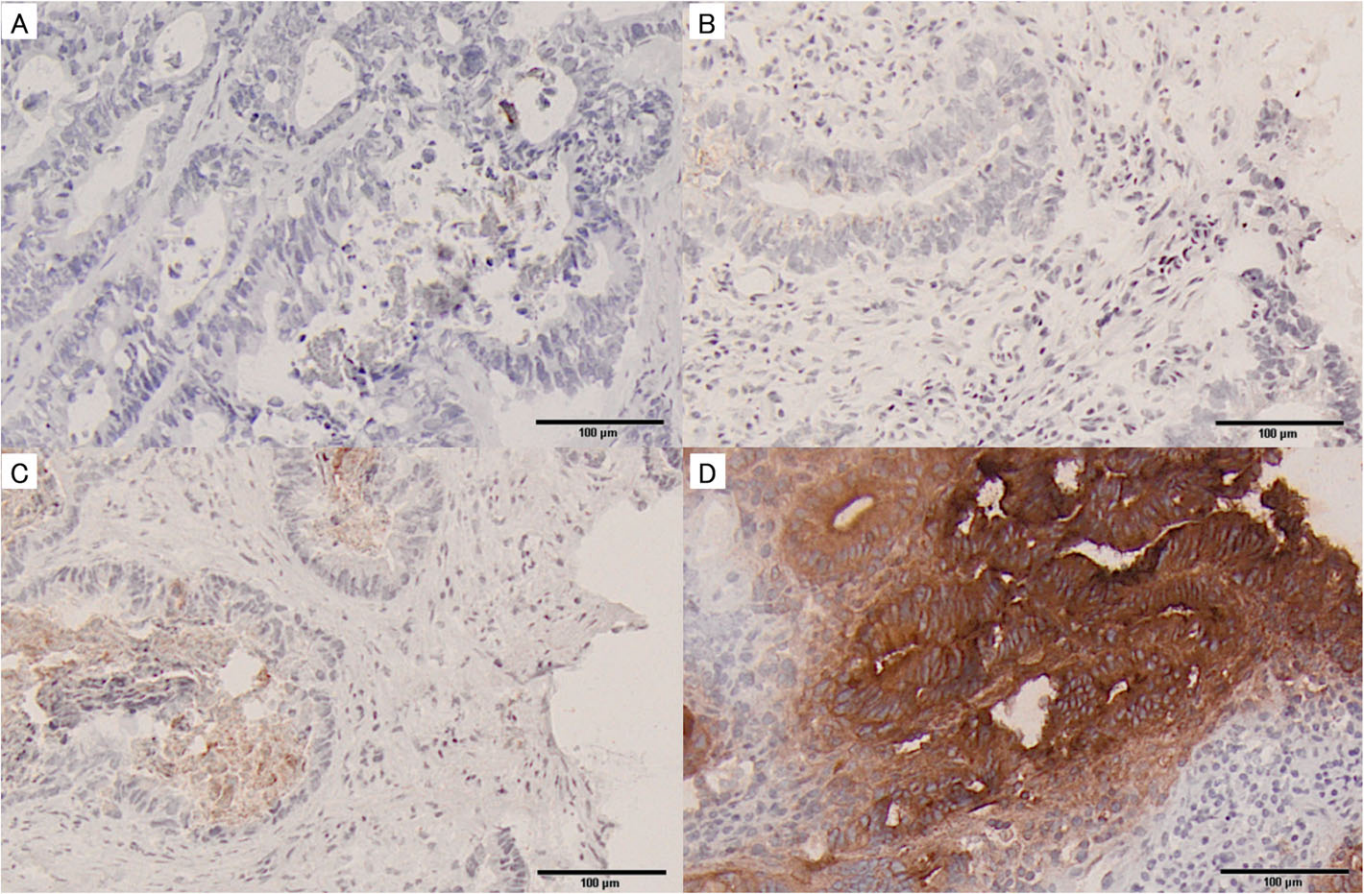
Immunohistochemical analyses of TMEM180 expression in colorectal cancer. **a** Immunonegativity. **b** 1+ reactivity intensity. **c** 2+ reactivity intensity. **d** 3+ reactivity intensity. Original magnification: × 100

Immunohistochemical evaluation was performed independently by five reviewers who were blinded to the clinicopathological details and clinical outcomes. All immunostained tissues were projected on the screen, and each case was discussed the following scoring by 5 reviewers at the same time. Both the percentage of positive cells and staining intensity were determined. Intensity of TMEM180 expression was scored as negative = 0, low = 1, moderate = 2, or strong = 3 (Fig. 1), and the percentage of positive cells among neoplastic cells was scored as 0% = 0, 1–10% = 1, 11–50% = 2, or 51–100% = 3. These two scores were multiplied to yield an IHC score, and a score of 2 or higher was considered as high TMEM180 expression after the ROC analysis was performed to determine the optimal cut off.

### Cells and cell culture

The human colon cancer cell line SW480 was purchased from the American Type Culture Collection (Manassas, VA, USA). TMEM180-knockdown SW480 cells were established according to our previously reported protocol [5, 7].

### Animal model, tumour-initiating activity, and anti-tumour activity

All experimental protocols were approved by the Committee for Animal Experimentation of the National Cancer Center. These guidelines meet the ethical standards required by law and comply with the guidelines for the use of experimental animals in Japan.

For evaluation of tumour-initiating activity, the mice were inoculated subcutaneously in the left flank with SW480-WT/−KD cells.

### Statistical analysis

Categorical variables were reported as numbers or percentages of patients, and analyzed by Chi-square or Fisher’s exact test. Quantitative variables were reported as median and interquartile ranges and analyzed with the Mann–Whitney U test. Survival curves of DFS and CSS were plotted according to the Kaplan–Meier method, and the generalized log-rank test was used to compare the survival curves. Univariate analyses of prognostic factors associated with DFS and CSS were performed using the Cox proportional hazards model. The Cox proportional hazards model was used to calculate hazard ratio (HR) and 95% confidential interval (CI) for low and high TMEM180. HR and 95% CI were calculated after adjustment for age, gender, tumour location, preoperative CEA level, tumour size, histological type tumour invasion, lymph node status, and adjuvant chemotherapy. For both the Kaplan–Meier method and Cox proportional hazards model, patients for whom more than 5 years of information were available were censored at 5 years post-diagnosis. All statistical analyses were performed using SPSS 22.0 (SPSS, Chicago, IL, USA), and the findings were considered statistically significant at *p* < 0.05.

## Results

The clinicopathological demographics of the patients are shown in Table 1. The median age of the patients at the time of surgery was 63.0 years (56.0–70.0 years). The study included 86 male and 71 female patients, with a median follow-up of 66.0 months (51.5–84.0 months). In total, 83 patients (52.9%) had colonic carcinoma, whereas 74 patients (47.1%) had rectal carcinoma; 125 of all patients (79.6%) received adjuvant chemotherapy.

**Table 1:** Clinicopathological features of 157 patients with stage III colorectal cancer

	All, *n* = 157 (%)
Age, years	63.0 (56.0–70.0)
Gender	
Female	71 (45.2)
Male	86 (54.8)
Location	
Colon	83 (52.9)
Rectum	74 (47.1)
Preoperative CEA level	
≤ 5 ng/ml	89 (56.7)
> 5 ng/ml	68 (43.3)
Histological type	
Well- or moderately differentiated	150 (95.5)
Poorly differentiated	7 (4.5)
Tumour invasion	
T1	2 (1.3)
T2	25 (15.9)
T3	108 (68.8)
T4	22 (14.0)
Lymph node status	
N1a	111 (70.7)
N1b	6 (3.8)
N1c	4 (2.6)
N2a	24 (15.3)
N2b	12 (7.6)
Stage	
IIIA	22 (14.0)
IIIB	120 (76.4)
IIIC	15 (9.6)
Adjuvant chemotherapy	
No	32 (20.4)
Yes	125 (79.6)
Follow-up, months	66.0 (51.5–84.0)

The intensity of TMEM180 staining and percentage of TMEM180-positive cells are shown in Table 2. TMEM180 expression was detected in 73 cases (46.5%): 1+ in 30 cases (19.1%), 2+ in 22 cases (14.0%), and 3+ in 21 cases (13.4). The percentage of TMEM180 was also evaluated: 84 cases (53.5%) were negative, 15 (9.6%) were 1–10% positive, 33 (21.0%) were 11–50% positive, and 25 (15.9%) were 51–100% positive.

**Table 2:** Intensity of TMEM180 staining and percentage of TMEM180-positive cells

Intensity of TMEM180 staining	
0	84 (53.5)
1+	30 (19.1)
2+	22 (14.0)
3+	21 (13.4)
Percentage of TMEM180-positive cells	
0	84 (53.5)
≤ 10	15 (9.6)
11–50	33 (21.0)
50<	25 (15.9)

**Fig. 2:**
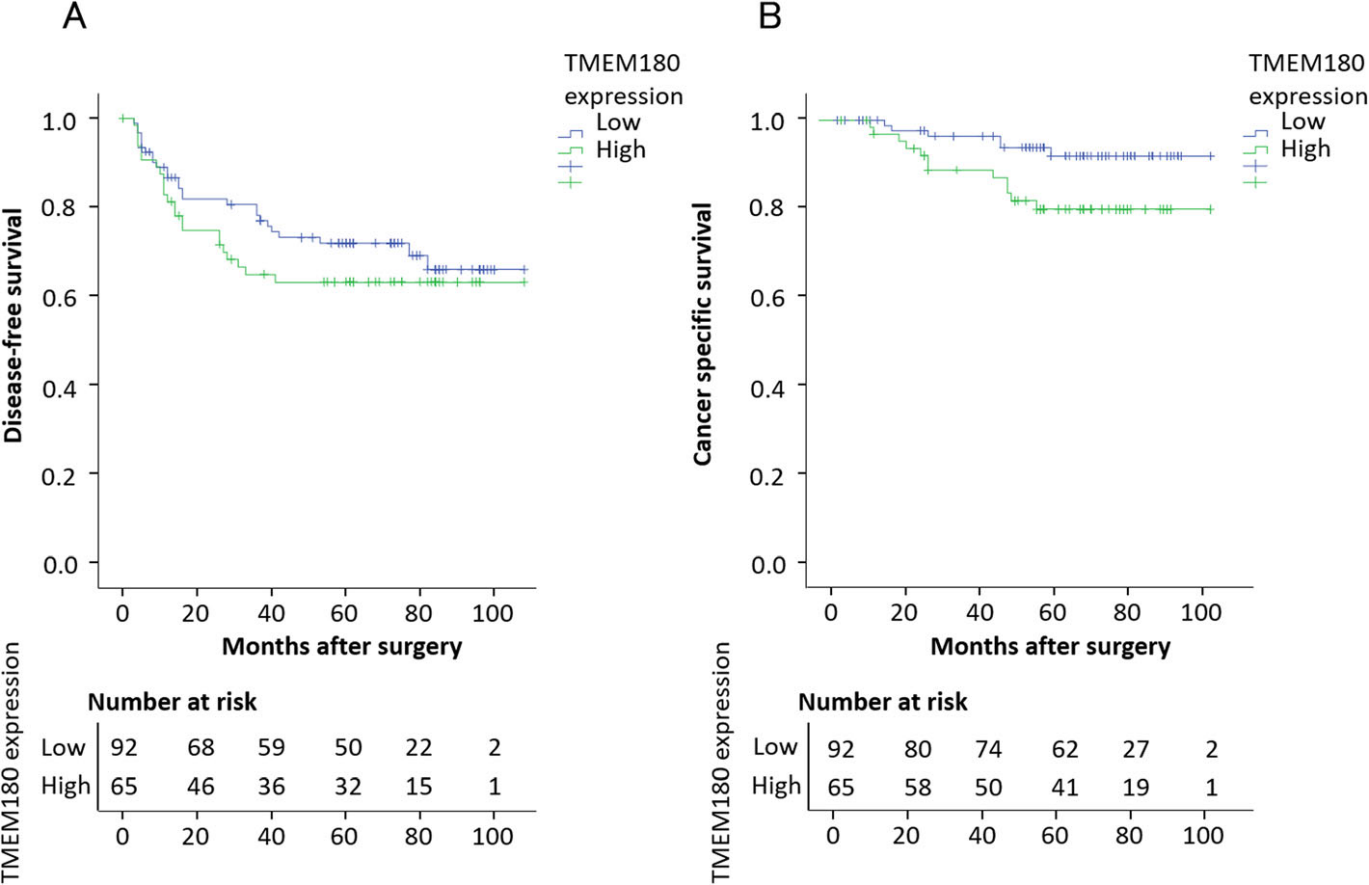
Disease-free survival (DFS) and cancer specific survival (CSS) curves of CRC generated by the Kaplan–Meier method and compared by log-rank test. **a** Patients with high TMEM180 expression exhibited poorer DFS than those with low TMEM180 expression, but the difference was not significant (*p* = 0.324). **b** Patients with high TMEM180 expression exhibited significantly poorer CSS than those with low TMEM180 expression (*p* = 0.028)

In total, 92 cases had low TMEM expression and 65 had high TMEM180 expression. TMEM180 expression was independent of any clinicopathological feature except gender (Table 3). The Kaplan–Meier curves for DFS and CSS of stage III CRC patients, categorized according to the level of TMEM180 expression. Patients with high TMEM180 expression exhibited poorer DFS than those with low TMEM180 expression, but the difference was not significant (*p* = 0.324) (Fig. 2a). Patients with high TMEM180 expression exhibited significantly poorer CSS than those with low TMEM180 expression (*p* = 0.028) (Fig. 2b). In terms of DFS, except for lymph node status (*p* = 0.049), prognosis associated with stage III CRC did not differ significantly according to age, gender, tumour location, preoperative CEA level, tumour size, histological type tumour invasion, adjuvant chemotherapy, or TMEM180 expression. On the other hand, in terms of CSS, patients whose tumours were high TMEM180 expression had a significantly worse prognosis than those whose tumours were low TMEM180 expression (*p* = 0.036) (Table 4). There was no significant difference according to age, gender, tumour location, preoperative CEA level, tumour size, histological type tumour invasion, lymph node status, or adjuvant chemotherapy. HR, calculated using the Cox proportional hazards model for TMEM180 expression after adjustment for other factors, is shown in Table 5. For DFS, HR in high-TMEM180 cases was 1.449 (95% CI = 0.802–2.619) higher than in low-TMEM180 cases, but the difference was not significant (*p* = 0.219). For CSS, HR in high-TMEM180 cases was 3.302 (95% CI = 1.088–10.020), significantly higher than in low-TMEM180 cases (*p* = 0.035).

**Table 3:** Correlation between high or low TMEM180 expression and clinicopathological features

	Low, *n* = 92 (%)	High, *n* = 65 (%)	p
Age, years			
≤ 65	59 (64.1)	41 (63.1)	0.892
> 65	33 (35.9)	24 (36.9)	
Gender			
Female	35 (38.0)	36 (55.4)	0.032
Male	57 (62.0)	29 (44.6)	
Location			
Colon	48 (52.2)	35 (53.8)	0.836
Rectum	44 (47.8)	30 (46.2)	
Preoperative CEA level			
≤ 5 ng/ml	55 (59.8)	34 (52.3)	0.352
> 5 ng/ml	37 (40.2)	31 (47.7)	
Tumour size			
≤ 5 cm	39 (42.4)	23 (35.4)	0.376
> 5 cm	53 (57.6)	42 (64.6)	
Histological type			
Well- or moderately differentiated	89 (96.7)	61 (93.8)	0.314
Poorly differentiated	3 (3.3)	4 (6.2)	
Tumour invasion			
< T3	15 (16.3)	12 (18.5)	0.724
T3≤	77 (83.7)	53 (81.5)	
Lymph node status			
N1	72 (78.3)	49 (75.4)	0.673
N2	20 (21.7)	16 (24.6)	
Adjuvant chemotherapy			
No	19 (20.7)	13 (20.0)	0.920
Yes	73 (79.3)	52 (80.0)	
Follow up, months	68.0 (52.3–84.8)	68.0 (53.5–84.5)	0.531

**Table 4:** Univariate analyses of the prognostic factors for disease-free survival (DFS) and cancer specific survival (CSS)

Variable	n	5-year DFS (%)	HR	95% CI	p	5-year CSS (%)	HR	95% CI	p
Age, years					0.463				0.318
≤ 65	100	72.0	1.000	0.698–2.204		91.0	1.000	0.634–4.079	
> 65	57	66.7	1.240			87.7	1.608		
Gender					0.808				0.412
Female	86	70.9	1.000	0.612–1.880		91.9	1.000	0.583–3.742	
Male	71	69.0	1.072			87.3	1.476		
Tumour location					0.052				0.768
Colon	83	75.9	1.000	0.994–3.110		90.4	1.000	0.456–2.896	
Rectum	74	63.5	1.759			89.2	1.149		
Preoperative CEA level					0.957				0.776
≤ 5 ng/ml	89	69.7	1.000	0.541–1.693		89.9	1.000	0.445–2.960	
> 5 ng/ml	68	70.6	0.957			89.7	1.148		
Tumour size					0.632				0.789
≤ 5 cm	62	66.1	1.000	0.496–1.531		88.7	1.000	0.340–2.67	
> 5 cm	95	72.6	0.872			90.5	0.878		
Histological type					0.605				0.678
Well- or moderately differentiated	150	70.7	1.000	0.423–4.378		90.0	1.000	0.204–11.534	
Poorly differentiated	7	57.1	1.361			85.7	1.534		
Tumour invasion					0.906				0.871
< T3	27	70.4	1.000	0.712–1.467		92.6	1.000	0.321–3.827	
T3≤	130	70.0	1.022			89.2	1.108		
Lymph node status					0.049				0.993
N1	107	73.6	1.000	1.003–3.311		88.4	1.000	0.572–1.739	
N2	36	58.3	1.822			94.4	0.997		
Adjuvant chemotherapy					0.717				0.122
No	32	71.9	1.000	0.554–2.361		87.5	1.000	0.157–1.244	
Yes	125	69.6	1.144			90.4	0.442		
TMEM180 expression					0.328				0.036
Low	92	73.9	1.000	0.755–2.320		94.6	1.000	1.071–7.603	
High	65	64.6	1.323			83.1	2.853

**Table 5:** Multivariate analyses of prognostic factors for disease-free survival (DFS) and cancer specific survival (CSS)

Variable	DFS			CSS		
	HR ^a^	95% CI	p	HR ^a^	95% CI	p
TMEM180			0.219			0.035
Low	1.000	0.802–2.619		1.000	1.088–10.020	
High	1.449			3.302		

*HR* hazard ratio, *CI* confidence interval

^a^Adjusted for age, gender, tumour location, preoperative CEA level, tumour size, histological type tumour invasion, lymph node status, and adjuvant chemotherapy

**Fig. 3:**
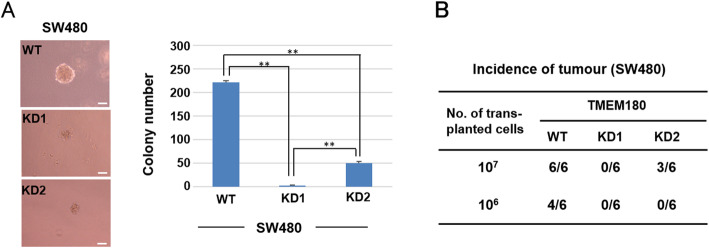
Relationship between TMEM180 expression and the tumourigenesis of SW480 CRC cells. **a** In an assay examining in vitro colony-forming activity in soft agar, SW480-WT cells formed colonies, whereas KD1 and KD2 did not. **b** The in vivo tumour-initiating activity of SW480 cell lines was positively correlated with the level of TMEM180 expression

We then sought to determine whether TMEM180 is involved in tumourigenesis. In an assay examining in vitro colony-forming activity in soft agar, SW480-WT cells clearly formed colonies, but neither KD1 nor KD2 cells did (Fig. 3a). The in vivo tumour-initiating activity of SW480 cell lines was positively correlated with the level of TMEM180 expression (Fig. 3b). Therefore, loss of TMEM180 expression correlates with a loss of the tumourigenesis of SW480 CRC cells.

## Discussion

This is the first study of the relationship between TMEM180 expression in CRC human tissues and patient survival. We used immunohistochemical analysis to monitor the expression of TMEM180 in human stage III CRC. We found that TMEM180 expression correlated significantly with patient survival; specifically, high TMEM180 expression was significantly correlated with poor CSS. By contrast, high expression of TMEM180 was not significantly correlated with poor DFS, although higher expression had a tendency with shorter DFS. Furthermore, high TMEM180 expression was an independent prognostic factor for poor CSS. TMEM180 expression in SW480 cells was positively correlated with anchorage-independent colony formation, and tumourigenesis.

One of the most important pathological characteristics of solid tumours is hypoxia. In a previous study, we showed that TMEM180 expression in CRC cells was up-regulated by hypoxia [5]. Hypoxia is clinically associated with poor prognosis because it can adversely affect the outcome of radiotherapy and chemotherapy [8–11]. Several studies have reported that a hypoxic environment or stresses such as chemotherapy induce HIF-1 expression, followed by T cell anergy and increased dissemination of cancer cells [12, 13]. However, we did not closely examine its relationship to expression such as hypoxia inducible factor (HIF)-1 and carbonic anhydrase IX in the present TR study. Although we are investigating the function of TMEM180 using several methods including proteomics, next generation sequence, metabolomics and oxygen consumption, there is no doubt that the expression of this molecule is increased by hypoxia in cancer cells. Indeed, we found that TMEM180 possesses 10 hypoxia-responsive element (HRE) consensus sequences in its promoter region [5]. We consider that it should be clarified whether TMEM180 expression occurs in HIF-dependent or HIF-independent systems soon. In addition, we have reported that TMEM180 have 12 transmembrane domains and found that putative cation binding site of TMEM180MelB [6]. These results suggest that TMEM180 would act as a cation symporter. Very recently, we have reported that TMEM180 may cause variation in metabolites related to glycolysis [14]. We are going to identify the substrates that are transported by coupling with cations in order to clarify the relationship between TMEM180 expression and tumour growth. Although information is limited, our data suggest that TMEM180 is involved in tumour growth.

Our data indicate that specific treatment and rigorous follow-up are needed for CRCs with high TMEM180 expression. Cetuximab is an anti-EGFR mAb that is widely used in patients with CRC. However, the use of cetuximab is limited to CRC patients with wild-type KRAS and has serious skin toxicity due to the high expression of EGFR in normal skin. On the other hand, anti-TMEM180 mAb exerts a significant antitumour effect in CRC xenografts with mutant KRAS [5]. Since this is very important in constructing the design of future clinical trials, we are conducting experiments to compare the effects of anti-TMEM180 antibody and anti-EGFR antibody on several patients derived xenografts of CRC with or without KRAS mutation. In regard to toxicity, a previous IHC study by our group revealed no TMEM180 expression in any major organ, including skin, brain, heart, lung, liver, intestine, and kidney. Moreover, our study of TMEM180-KO mice did not reveal any embryonic, neonatal, or postnatal lethality [5]. According to these data, we anticipate that unpredictable adverse effects are unlikely to occur in a clinical trial of anti-TMEM180 mAb. Our mAb is now being processed according to Good Manufacturing Practice (GMP) principles for testing in a first-in-human clinical trial.

This study had some limitations. First, it was a retrospective study at a single institution and had a small sample size. Because only stage III cases were targeted, other stages cases were not analyzed. Accordingly, further analysis using a larger population is needed to clarify the prognostic significance of CRC patients with TMEM180 expression. Second, we used frozen tissue specimens stored for long periods of time, and it is possible that the tissues had deteriorated. Third, we only used SW480 cell lines in this study and reported the positively correlation with anchorage-independent colony formation and tumourigenesis. As previously reported, the xenografts model showed the most significant effect in therapeutic experiments with the antibody of SW480 cell lines [5]. However, other recent basic experiments have confirmed that knockout cells of other colorectal cancer cells, LoVo and DLD-1, have a clearly reduced proliferative capacity compared to their parent strains. However, despite these limitations, our results suggested that expression of TMEM180 is an important prognostic factor in stage III CRC patients. Undoubtedly, a prospective study is needed to confirm the conclusion. We are now developing an antibody suitable for IHC using FFPE samples; this would allow us to perform a translational study using larger numbers of archival samples.

## Conclusion

We found that TMEM180 expression was significantly associated with poor CSS. Furthermore, high TMEM180 expression was an independent prognostic factor for poor CSS. We showed that TMEM180 expression is involved in tumour proliferation. We believe that these fundamental data warrant further basic and translational studies of TMEM180, and its mAb, for development of therapeutics against CRC.

## Data Availability

The datasets used and/or analysed during the current study available from the first author TS on reasonable request.

## Abbreviations

CRC: ColoRectal Cancer
TMEM 180: TransMEMbrane protein 180
mAb: Monoclonal Antibody
CSS: Cancer Specific Survival
DFS: Disease-Free Survival
HIC: ImmunoHistoChemistry
HR: Hazard Ratio
CI: Confidential Interval
HRE: Hypoxia-Responsive Element
HIF: Hypoxia Inducible Factor
